# Unusually Long Palindromes Are Abundant in Mitochondrial Control Regions of Insects and Nematodes

**DOI:** 10.1371/journal.pone.0000110

**Published:** 2006-12-20

**Authors:** K. P. Arunkumar, Javaregowda Nagaraju

**Affiliations:** Laboratory of Molecular Genetics, Centre for DNA Fingerprinting and Diagnostics, Nacharam, India; Temasek Life Sciences Laboratory, Singapore

## Abstract

**Background:**

Palindromes are known to be involved in a variety of biological processes. In the present investigation we carried out a comprehensive analysis of palindromes in the mitochondrial control regions (CRs) of several animal groups to study their frequency, distribution and architecture to gain insights into the origin of replication of mtDNA.

**Methodology/Principal Findings:**

Many species of Arthropoda, Nematoda, Mollusca and Annelida harbor palindromes and inverted repeats (IRs) in their CRs. Lower animals like cnidarians and higher animal groups like chordates are almost devoid of palindromes and IRs. The study revealed that palindrome occurrence is positively correlated with the AT content of CRs, and that IRs are likely to give rise to longer palindromes.

**Conclusions/Significance:**

The present study attempts to explain possible reasons and gives in silico evidence for absence of palindromes and IRs from CR of vertebrate mtDNA and acquisition and retention of the same in insects. Study of CRs of different animal phyla uncovered unique architecture of this locus, be it high abundance of long palindromes and IRs in CRs of Insecta and Nematoda, or short IRs of 10–20 nucleotides with a spacer region of 12–14 bases in subphylum Chelicerata, or nearly complete of absence of any long palindromes and IRs in Vertebrata, Cnidaria and Echinodermata.

## Introduction

A DNA palindrome is a unique case of inverted repeats (IRs) [1] where a segment of nucleotides is immediately followed by its reverse complement. Palindromes are involved in a variety of biological processes, for example acting as recognition sites for bacterial restriction enzymes to cut foreign DNA [2]. They also play important role in DNA replication and gene regulation [3], [4]. IRs flanking the origin of DNA replication with the potential of forming single-stranded stem-loop cruciform structures have been reported to be essential for replication of the circular genomes of many prokaryotic and eukaryotic systems [5]. Several studies have reported the existence of high concentrations of palindromes in proximity to the replication origins of viruses [6]–[8]. The local two-fold symmetry created by the palindrome is thought to provide binding site for DNA-binding proteins that are often dimeric. Such double binding markedly increases the strength and specificity of the interaction [9]. These regions have been associated with replication origins of a few herpesviruses, bacterial plasmids, etc. In an earlier study [8] it was demonstrated that by looking for palindrome clusters, along with other features such as clusters of close repeats and close inversions on the nucleotide sequence, it is possible to fish out regions from a genome that are likely to harbor replication origins. Also, perfect palindromes, quasi-palindromes and IRs separated by spacers, all have the potential to form secondary structures and are known to cause genetic instability in *Escherichia coli* [10], yeast [11], and in mouse [12], [13].

Metazoan mitochondrial DNA (mtDNA) is a closed-circular, double-stranded molecule, ranging in size from 15 to 20 kb [14]. It contains a distinct replication origin on each of the DNA strands. Initiation of mtDNA replication is controlled by the interaction between nuclear-encoded proteins and regulatory sequences existing on the mtDNA [15]–[17]. The non-coding region of the mitochondrial genome in animals called the “control region” (CR) is believed to control the transcription and replication of mtDNA. In vertebrates the CR has been shown to contain promoters for transcription initiation and the origin of heavy-strand DNA replication [15]. In insects this region is usually called “A-T rich region” [18]. Information on palindromes and IRs in mitochondrial CRs and their role in origin of replication is scanty and needs further investigation. In the present study we have attempted to dissect the architecture of the origin of replication of mtDNA by analyzing CRs of several animal phyla and also carried out comprehensive analysis to study the frequency and distribution of palindromes and IRs of eight animal groups.

## Methods

### Source of CR sequences

From NCBI, sequences of mitochondrial CRs were queried in 12 most studied animal phyla (Porifera, Cnidaria, Platyhelminthes, Nemertina, Rotifera, Nematoda, Brachiopoda, Mollusca, Annelida, Arthropoda, Echinodermata and Chordata) and were downloaded from different phyla/classes/orders separately wherever necessary, by carrying out a boolean search using combination of different keywords. For example, to download the CR sequences of lepidopteran species, we used keywords like lepidoptera AND control region or lepidoptera AND D loop. Each sequence description was manually checked to ensure that we downloaded CR sequences only.

**Figure 1:**
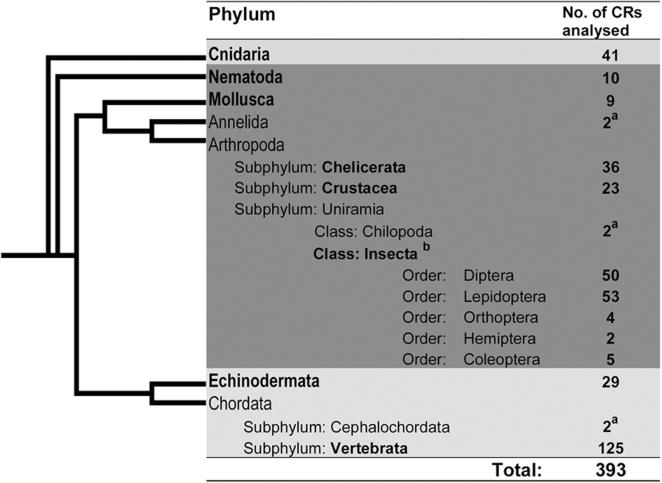
Number of species analysed for the presence of palindromes and inverted repeats in CRs. In a few phyla, significant number of CRs was not available. **^a^**These sequences were not used for analysis due to small sample size. **^b^**CRs from all the orders clubbed together and treated as one animal group ‘Insecta’. Phyla in which <10% of the species harboring palindromes in their CRs are represented in light gray background and >10% in dark gray background. The phylogenetic tree is only a schematic representation and is not according to distance

To study the abundance of palindromes and IRs in subphyla, classes and orders of the phyla Arthropoda and Chordata, CR sequences were downloaded separately from three subphyla Chelicerata, Crustacea and Uniramia of phylum Arthropoda. Subphylum Uniramia was further divided into Chilopoda and Insecta. Many CR sequences have been reported in class Insecta. Therefore, we further binned Insecta CRs based on the taxonomic order of origin. From phylum Chordata we extracted CRs from two important subphyla, Cephalochordata and Vertebrata. Details of the sample size are given in figure 1.

Finally, based on number of sequences available in each phylum we divided the data into eight animal groups namely Cnidaria, Nematoda, Mollusca, Echinodermata, Chelicerata, Crustacea, Insecta and Vertebrata (Figure 1; see Supplementary File S1 for complete list of species names and other details).

### Mining palindromes in control regions

**Figure 2:**
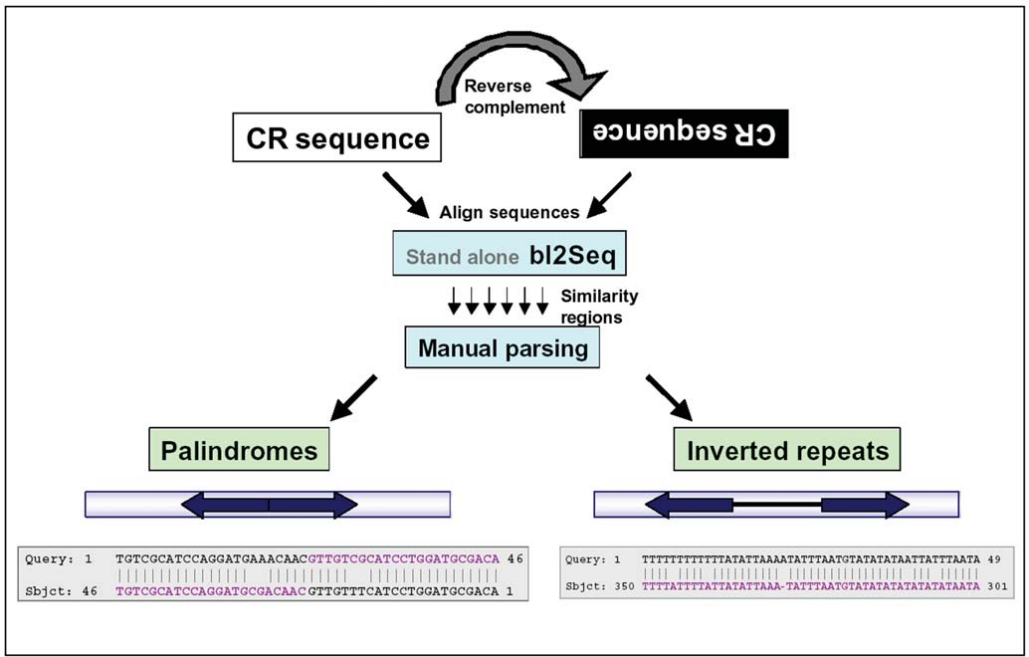
Schema of palindrome and inverted repeats mining, adapted in this study. Example of a typical palindrome and inverted repeat unit is given at the bottom. Bars with arrows represent CR sequences. In alignments where subject and query are same but reverse complements, were considered as palindromes. In alignments where subject and query are separated by a spacer region of >13 bases, were considered as IRs.

A novel strategy to identify palindromes and IRs of different lengths was devised (Figure 2). For this purpose we adopted ‘bl2seq’ (align 2 sequences) program available in standalone BLAST package of NCBI [19]. A perl script was written, which takes the sequence as query and reverse complement of the same sequence as the subject, and searches for the stretch of similar sequence between them using ‘bl2seq’ program. Pair-wise alignments with more than 70% match were printed to the file. Default parameters of ‘bl2seq’ were used to carry out BLAST. If there is a spacer region of >13 bases between the inverted repeat regions, then that sequence was considered as an IR. This program was executed using multi FASTA files containing CR sequences as input and output files were manually parsed to extract the desired information. The output file was manually verified for confirming the selection of palindromes and tabulated in Microsoft excel data sheets. The program was found to be efficient in identifying palindromes and IRs of longer lengths, allowing a certain level of mismatch. The perl script used for the analyses can be downloaded from www.cdfd.org.in/lmgpgms.html. We did not use ‘Palindrome’ program developed by EMBOSS, since the output did not give the length of sequence and was difficult to make out whether the output alignment is a palindrome or IR sequence. Also, there was no option for setting minimum percent similarity allowed.

### Analysis of palindromes and inverted repeats

The perl program gave output of BLAST alignments. From these alignments information on presence, number and length of palindromes and IRs was sorted (Figure 2). Sequences of ≥20 bases were considered as palindromes in order to avoid restriction enzyme recognition sites. The data were tabulated to calculate the frequency of palindromes and IRs in different phyla, classes and orders. Only eight animal groups (Figure 1) were used for the analysis of palindromes and IRs, since the number of CR sequences was not adequate for the remaining animal groups. Since only 5% of the vertebrates possessed palindromes and IRs of ≥20 bases, we also analyzed palindromes and IRs of 10–20 bases for length variation within closely related organisms to get clues for their departure from subphylum Vertebrata. We also looked into subphylum Chelicerata where small IRs of 12–14 bases were present in more than half of the CRs studied.

To find out the correlation of AT richness with the frequency of occurrence of palindromes in CRs we estimated AT content of all CRs using a C program written in-house. The C program can be downloaded from www.cdfd.org.in/lmgpgms.html. AT content of all the reported complete mitochondrial sequences of different animal phyla was also calculated to draw correlation if any, between occurrence of palindromes in CRs and AT content of complete mitochondrial sequences. Further, statistical analyses were carried out to estimate the abundance of palindromes and IRs in different animal groups and to establish relationship between AT content and palindrome occurrence in CRs. All these analyses were carried out in Microsoft excel data sheets. AT content of all the CR sequences analyzed can be found in Supplementary File S1. Unpaired t-test was carried out on the AT content of all 8 animal groups, to find out whether the AT content values are statistically different between animal groups. In the present study more emphasis is given to insect and vertebrate species as more CR sequences are reported in these animal groups, which led us to address several basic questions like why insect CRs are rich in palindromes and IRs.

## Results and Discussion

Evidence accrued so far suggests that mitochondria, once existed as free-living bacteria, were taken up by primitive ancestors of eukaryotic cells in an arrangement termed ‘endosymbiosis’ [20]. Till recently it was believed that replication mechanism in mtDNA is conserved and resembles that of plasmid replication. But recent reports suggest that replication mechanism of mtDNA varies among different animal phyla [21], [22]. Metazoan mtDNA codes for 13 or 14 proteins involved in the electron transport chain, 2 rRNA subunits, and 22 tRNA molecules. It contains a distinct replication origin on each of the DNA strands. Mammalian and insect mtDNAs maintain two separate and distinct origins of replication for unidirectional synthesis of each strand of the genome; however, location of these origins is not same in these two animal groups.

### Palindrome occurrence in different animal phyla

**Figure 3:**
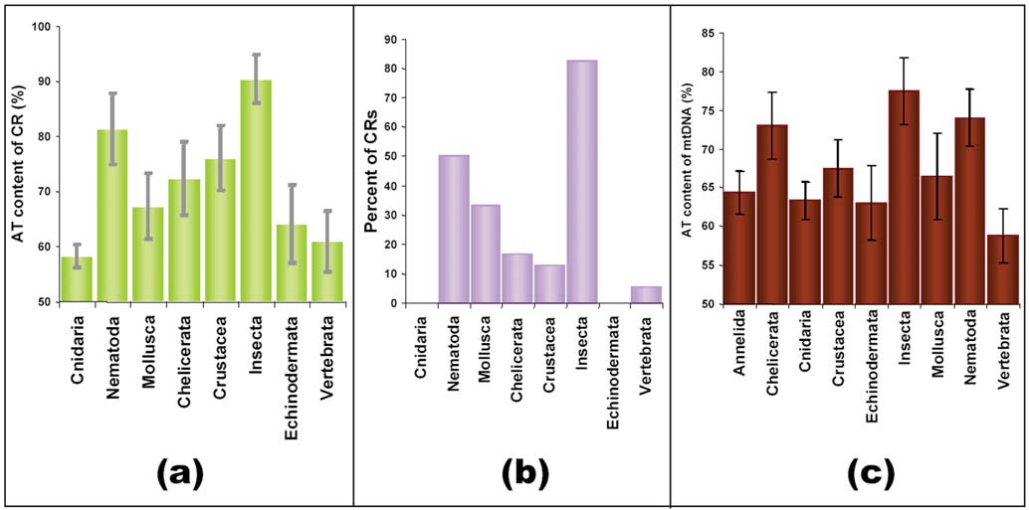
(a) Mean AT content of CR (%) of mitochondrial CRs (n = 393) and (b) % of mitochondrial CRs having palindromes in different animal groups (n = 387). (c) Mean AT content of CR (%) of complete mitochondrial sequences of different animal groups (n = 201). Data was obtained from analysis of 8 animal groups.

A total of 393 CR sequences of eight animal groups (Figure 1) was analysed for the distribution and frequency of palindromes and IRs. The analysis revealed interesting features. Palindromes were preponderant in invertebrates than vertebrates. Among invertebrates, more than half of the species in Insecta (85%) and Nematoda (50%) were found to possess palindromes, whereas 35, 20 and 15 percent of the species of Mollusca, Chelicerata and Crustacea respectively, had palindromes. Echinoderms and cnidarians were completely devoid of palindromes and IRs. On the other hand, only 5 percent of vertebrates contained palindromes and mammals were characterized by their complete absence (Figure 3b).

Comparative genomics studies suggested that the mitochondrion is monophyletic in origin [20] and the original mitochondrial endosymbiont has evolved independently in anaerobic and aerobic environments that are inhabited by diverse eukaryotic lineages. The evolution of various kinds of DNA motifs in CRs such as palindromes, IRs and other recognition elements in different animal phyla, appeared to have occurred independently after the divergence of different animal phyla leading to variation in number of palindromes and IRs.

In vertebrate mitochondrial CRs analysed in the present study, palindromes were found in a few avians (*Gallus* sps. and *Syrmaticus ellioti*), two fish species (*Apeltes quadracus*, *Cyprinodon bovines*) and a reptile (*Teratoscincus keyserlingii*). IRs were found only in one species, *T. keyserlingii* out of 125 vertebrates analysed.

Subphylum Chelicerata is unique in its composition of CR. Nineteen out of 36 CRs analysed in this subphylum harbored short IRs of 10–20 nucleotides with a spacer region of 12–14 nucleotides. Except for *Acropora longicyanthus* with a short palindrome of 14 nucleotides, phylum Cnidaria was completely devoid of palindromes and IRs.

### Palindromes in mitochondrial CRs of arthropods

**Figure 4:**
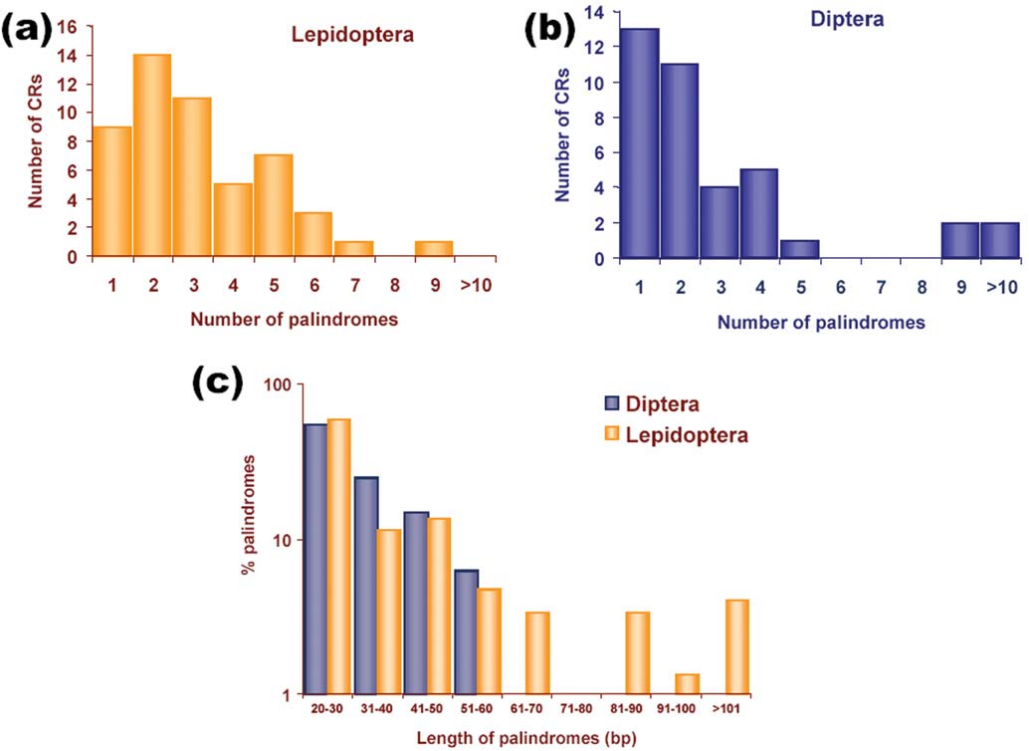
Distribution of palindromes in CRs of (a) Lepidopterans (n = 53) and (b) Dipterans (n = 50). Number of CRs is plotted against the number of palindromic DNA stretches they contain. Many CRs were having ≥2 stretches of palindromes in Lepidopterans. Dipteran CRs possess less number of palindromes when compared to Lepidopterans. (C) Distribution of palindrome lengths in dipteran (n = 82) and lepidopteran (n = 150) CRs. Palindromes falling to each class interval (20–30 to ≥100 bp) were grouped. Number of palindromes in each class was converted to percent values to compare the length distribution between Diptera and Lepidoptera. These percent values were plotted in graph against palindrome length class intervals. In Diptera, 38 of 50 CRs had palindromes whereas, 51 of 53 CRs analysed of Lepidoptera had palindromes.

Among invertebrates, in class Insecta higher abundance of palindromes and IRs was observed. Two important orders Lepidoptera and Diptera were analysed further for frequency and distribution of palindromes and IRs. Mitochondrial CR of lepidopteran insects turned out to be ‘hotspots’ of palindromes and IRs. All but two (*Erebia oeme* and *Pyronia tithonus*), of 53 species analysed in this order, harbored palindromes. When compared to order Diptera, lepidopterans possessed higher number of palindromes in CRs. Out of 50 dipterans, 38 had palindromes (Figure 4a & b). We compared the number of palindromes per CR of dipteran and lepidopteran species. In dipterans CRs harboring only one palindrome were more as compared to lepidopterans which harbored more than one palindrome per CR (Figure 4a & b). To study the length distribution of palindromes in lepidopteran and dipteran mitochondrial CRs, the length of palindromes was analyzed. The CRs, which had ≥1 palindrome(s) were considered for the analysis. The results revealed that lepidopteran CRs had longer palindromes than those of dipterans (Figure 4c).

Although palindromes exist naturally in the DNA sequence of many organisms, it is difficult to maintain long palindromes because of their genetic instability stability as demonstrated in *E. coli* [23]. The instability of palindromes is attributed to a number of causes. First, palindromes may be deleted as a result of intermolecular or intramolecular recombination. Second, the deletion of palindromes may result from the formation of a cruciform structure and the subsequent processing by nucleases. Third, in the case of palindromes containing direct repeats, misalignment between the direct repeats may be stabilized by the formation of a hairpin or other DNA secondary structures [24]. In the present study we observed surprisingly long palindromes greater than 150 bp in three (*Epirrita christyi*, *Arethusana arethusa* and *Epirrita autumnata*) of the 53 lepidopterans studied. Even though palindromes are unstable due to several reasons listed above, they are retained in CR suggesting their possible involvement in replication initiation of lepidopteran mitochondrial genome. However, in dipterans we observed shorter palindromes of only up to 56 bp. None of the CR sequences analysed in the remaining phyla possessed palindromes greater than 150 bp. However, molecular function of these long palindromes in insect mtDNA needs further analysis.

### Palindromes occur in AT rich regions of CRs

**Figure 5:**
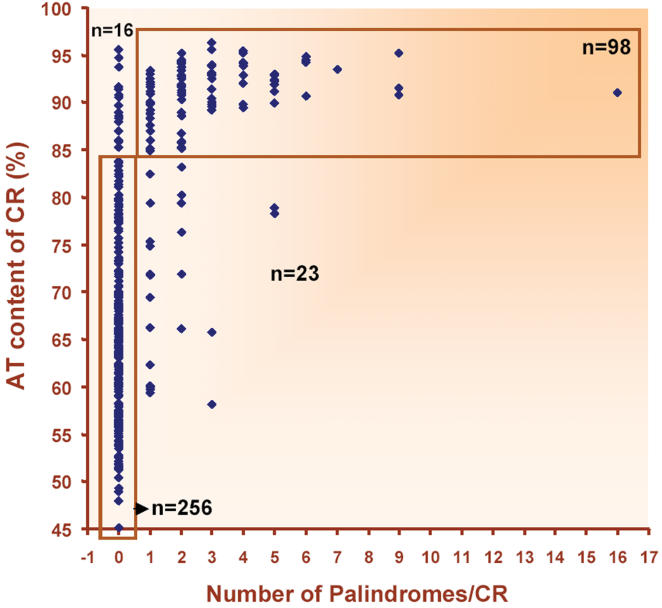
Distribution of number of palindromes per CR, plotted against AT content of CR (%). Majority of CRs with less than 85 percent AT content had no palindromes but most of the CRs with AT percent >85 possessed palindromes. Trend is that as the AT content increased, occurrence of palindromes in CRs also increased. Number of CRs analysed was 393.

Since insect CRs are AT-rich, it prompted us to establish correlation if any between AT content and palindrome occurrence. All CRs were pooled irrespective of the phyla to which the species belonged and analysed for presence or absence and number of palindromes in relation to AT content. Positive correlation between AT content and number of palindromes with correlation co-efficient of 0.89 was observed. Through these analyses we propose that palindromes originate in CRs with more than 85% AT content (Figure 5).

All animal groups except Mollusca and Echinodermata differed significantly (p<0.05) in their CR AT content (Supplementary File S2). Cnidaria, Echinodermata and Vertebrata, which harbored no or less number of palindromes and IRs in their CRs, have low AT content (an average of 58, 64, 61% respectively). Even though Chelicerata and Crustacea belong to phylum Arthropoda, to which insects belong, there is a marked difference in their AT content of CRs. Also, abundance of palindromes varies in these animal groups. Insects have high AT content and high occurrence of palindromes in the CRs, whereas Chelicerata and Crustacea have less AT and palindromes in their CRs (Figure 3a).

Analysis of complete mitochondrial genomes of Insecta and Nematoda also indicated the AT richness in their genomes (Figure 3c). Nematoda, Chelicerata and Insecta have an average AT content of more than 70%. Though Chelicerata mitochondrial genome is rich in AT content, only a few of the species harbored palindromes and IRs in the CR, inferring that AT content of complete mitochondrial genome has no relationship with the palindrome occurrence in CRs.

### Inverted repeats would give rise to long palindromes

The present study revealed surprisingly long palindromes in mitochondrial origin of replication. It is known that palindromes and IRs reside near the origin of replication in several bacteria, plasmids and viruses [25]. But these palindromes and IRs are invariably of shorter length (10–12 bp) unlike the palindromes reported in the present study.

**Figure 6:**
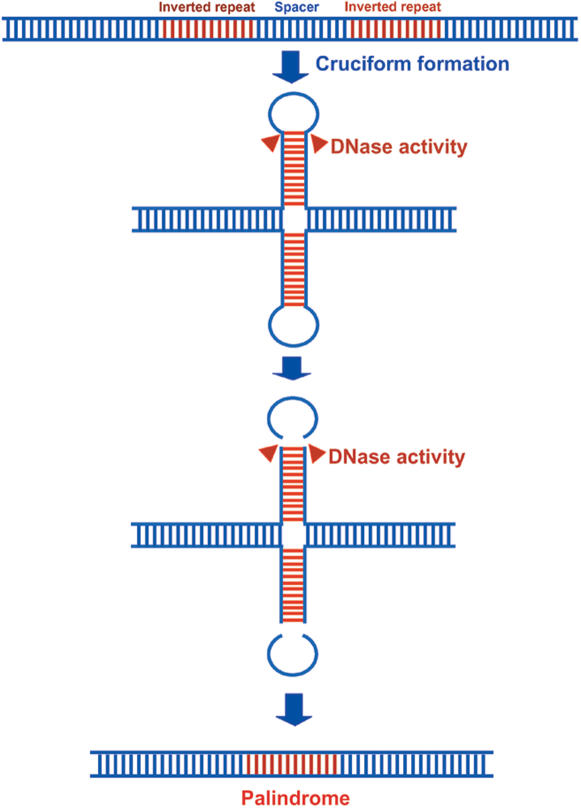
Schema showing possible mechanism of origin of long palindromes from IRs.

Long palindromic sequences are unstable since they are deleted at extremely high rates as reported in the case of *E. coli* [26], [27]. While short palindromes and IRs are usually much more stable; they can be associated with the breakpoints of deletion mutations as observed in *E. coli* [28], [29] and in mammalian cells [30]. Distantly separated long IRs are also prone to deletion in bacteria [31], [32]. In the present study palindromes were preponderant (in 120 CRs) over IRs (in 78 CRs). This shows that in CRs, palindromes are less prone to deletion unlike in *E. coli*. The presence of a fewer IRs led us to suspect that intervening regions between IRs would be deleted over a period of time resulting in formation of long palindromes. This can be explained in the following ways: i. when the DNA melts either during DNA replication or due to any other cellular activity, there would be intra-strand base-pairing in the regions of IRs leading to formation of hair-pin loop. The unpaired spacer regions between IRs may be eventually cleaved by DNases, as the tips of hairpin loops are sensitive to single-stranded nucleases [33]–[37] resulting in long palindromes (Figure 6). Higher abundance of palindromes as compared to IRs also supports this hypothesis. ii. Alternatively, occurrence of a DNA double-strand break near short inverted repeat sequences acts as a starting point in the formation of large DNA palindromes [38]–[43]. Bidirectional DNA replication would then convert the giant hairpin molecule to a palindrome.

However, we cannot exclude other factors, which are thought to lead to formation of long palindromes in CRs of insects, as in some cases they form as a result of a precisely regulated developmental program. For example, previous reports have shown the formation of long palindromes from short IRs by single strand annealing of IRs followed by removal of nonhomologous DNA and gap-filling DNA replication [37], [39], [43].

### Palindrome expansion and palindrome curing

Palindromes of 4–6 bp are implicated in bacterial chromosome and plasmid replication. Since mitochondria are thought to have originated from bacteria, the origin of replication also has a propensity to harbor palindromes. But palindromes present in CRs of insects are much longer than those present in bacterial origin of replication. Phylogenetic evidence derived from rRNA [44] and protein data [45] support the view that all mitochondrial genomes are descendents of a common proto-mitochondrial ancestor.

Due to varied evolutionary forces acting on different species, lower animal groups like Cnidaria and higher animal groups like echinodermates may be completely devoid of palindromes and, longer palindromes would have originated or expanded from already present smaller palindromes or IRs in some species of insects and nematodes, depending on the bio-physiochemical environment within the cell. Although the genetic role of mtDNA appears to be universally conserved, this genome exhibits remarkable variation in conformation and size, as well as in actual gene content, arrangement and expression [46] including palindrome occurrence in CRs.

The surprising absence of long palindromes and IRs in CR of vertebrate mtDNA implies that during the course of evolution, higher animals like chordates probably have adopted a slightly different mechanism of replication of mtDNA which does not require palindromes as a recognition motif in replication initiation. The variation in distribution of palindromes and IRs in different animal groups suggests that the mode of replication origin is quite different between different animal phyla. This is supported by the previous studies on CR of vertebrates and insects [21], [22].

The AT bias is generally observed in insect mitochondrial genomes, which ranges from 69.5 to 84.9% [47], [48] as against 53 to 66% in vertebrates. The strongest AT bias is found in the CR. The CR contains the origin of replication for the heavy strand in vertebrates [49] and both strands in Drosophila [50]. Mitochondrial gene order variation occurs both between and within animal phyla [51]. In insects, the CRs, which account for 80–95% AT, lack any apparent signals such as conserved sequence blocks, for the initiation of replication like those observed in vertebrates [52], [53]. These differences in replication initiation mechanism of vertebrates and insects probably hold the answer as to why CRs of some phyla are rich in palindromes and IRs.

The vertebrates were also found to be deficient in short palindromes. Of the 125 species examined, only 50 harbored short palindromes. Multiple sequence alignment of these short palindromic sequences showed contraction of a few palindromes, indicating that during course of evolution palindromes are lost gradually by reduction in size. Contraction of palindromes would be due to mutations at both ends of palindromes as evident from *Gallus* sps (Supplementary File S3).

### Why long palindromes in CRs of Insecta only?

**Figure 7:**
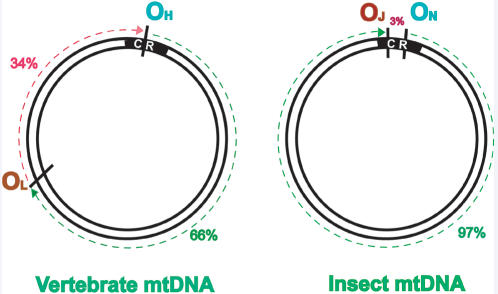
Illustration of origin of replication of vertebrate (heavy strand, O_H_; light strand, O_L_) and insect (minor strand, O_N_; major strand, O_J_) mtDNA. In vertebrates, the synthesis of L-strand is initiated from the O_L_, only when the synthesis of H-strand reaches O_L_, which is located about two third of the distance from O_H_. In insects, the replication origin for major strand (O_J_) is located 97% of the genomic distance away from the replication origin for minor strand (O_N_), that is, within the CR.

Metazoan mtDNA contains a distinct replication origin on each of the DNA strands. The position of the replication origin and mode of replication have been studied in detail in mammalian mtDNA and also recently in insects [21]. In mammals, the replication begins from the replication origin of H-strand (O_H_) and DNA synthesis proceeds unidirectionally. When the synthesis of H-strand reaches two-thirds of the genome, the synthesis of L-strand (O_L_) is initiated from the replication origin of the L-strand located two-thirds of the genomic distance away from the replication origin of H-strand [15], [16] (Figure 7). The previous observations indicate that regulatory sequences of mtDNA replication are different in invertebrate and vertebrate species and therefore suggest that the regulatory systems have changed through the evolution of animals [21].

Several regulatory sequences have been identified in the CR of the vertebrate mtDNA. These are present immediately upstream of the O_H_ and are suggested to be implicated in the initiation of the H-strand replication [54], [55]. These regulatory sequences are thought to be involved in generating the 3′ ends of the RNA primers, which are required for the DNA synthesis of the H-strand. Around the O_L_, the IR sequence of 10–12 bp, that could form a stem-loop configuration is conserved among vertebrate species and is also proposed to be required for the initiation of replication [54]. *In vitro* replication studies have suggested that the IR sequence serves as a recognition motif for mtDNA primase which provides a short RNA primer for L-strand synthesis, and DNA synthesis is initiated near the base of the stem-loop structure utilizing the 3′ ends of the RNA primer [56].

In insects, the leading and lagging strands are termed minor and major coding strands according to the relative numbers of the gene encoded on the respective DNA strand [57]. The replication origin for minor strand (O_N_) is located in the middle portion of CR. Synthesis of minor coding strand proceeds unidirectionally, and the major coding strand synthesis begins after 97% of the minor coding strand synthesis is completed [58], [59] (Figure 7). If the replication mode is similar between both insect and vertebrate mtDNAs, the replication origin for major strand (O_J_) must be located 97% of the genomic distance away from the replication origin for minor strand, that is, within the CR [21]. The fact that CR contains the O_H_ in vertebrates [49] and both O_N_ and O_J_ in insects [50], tempts us to speculate that occurrence of palindromes and IRs would aid in replication of insect mtDNA.

According to the strand-asynchronous, asymmetric model of vertebrate mtDNA, the replication of the L-strand is initiated when the synthesis of the H-strand passes beyond the L-strand origin, and template strand for the L-strand replication becomes single stranded. In insects O_J_ is located 97% genomic distance away from the O_N _that is, both O_J_ and O_N_ lie within the CR. This observation is consistent with the recent findings in Drosophila mtDNA that the free 5′ ends in the CR near the tRNAIle gene, exactly 97% genomic distance away from the O_N_ [21]. To draw similarity between O_J_ of insects and O_L_ of vertebrates, there should be presence of IRs in O_J_ like in O_L_ to form stem loop structure for the initiation of replication from O_J_. Indeed in the present study we have found palindromes and IRs in CRs of insects.

### Conclusions

This is a comprehensive report on the analysis of palindromes and IRs comprising 393 CRs from seven animal phyla. We are reporting that long palindromes and IRs are abundant in insect mitochondrial origin of replication. Lower animals like cnidarians and higher animal groups like chordates are almost devoid of long palindromes, where as many species of Arthropoda, Nematoda, Mollusca and Annelida harbor palindromes and IRs in their CRs (Figure 1). Here we have given the primary data on the effect of AT richness on palindrome occurrence and plausible reasons for origin of longer palindromes from short inverted repeats. Study of CRs of different animal phyla uncovered unique architecture of this locus, be it high abundance of long palindromes and IRs in CRs of Insecta and Nematoda, or short IRs of 10–20 nucleotides with a spacer region of 12–14 bases in subphylum Chelicerata or nearly complete of absence of any long palindromes and IRs in Vertebrata, Cnidaria and Echinodermata.

## Supporting Information

Supplementary File S1 Details of the control region sequences used in the present study
(0.10 MB XLS)

Supplementary File S2 P-values obtained by t-test performed on AT percent values of 8 animal groups.
(0.01 MB PDF)

Supplementary File S3 CLUSTAL X - Multiple sequence alignment of short palindromes found in vertebrates
(1.61 MB PDF)
