# Supplementary material for: Unusually Long Palindromes Are Abundant in Mitochondrial Control Regions of Insects and Nematodes
Source: PLoS One. 2006 Dec 20;1(1):e110. doi: 10.1371/journal.pone.0000110 (PMC1762429; doi:10.1371/journal.pone.0000110)
Supplement: Supplementary File S2 — P-values obtained by t-test performed on AT percent values of 8 animal groups. (0.01 MB PDF) [file pone.0000110.s002.pdf]

P-values obtained by t-test performed on AT percent values of 8 animal groups.

	Cnidaria	Nematoda	Mollusca	Echinodermata	Chelicerata	Crustacea	Insecta	Vertebrata
**Cnidaria**		<0.001	<0.001	<0.001	<0.001	<0.001	<0.001	<0.001
**Nematoda**			<0.001	<0.001	0.001	0.037	0.002	<0.001
**Mollusca**				**0.192**	0.043	0.002	<0.001	0.012
**Echinodermata**					<0.001	0.002	<0.001	0.030
**Chelicerata**						0.032	<0.001	<0.001
**Crustacea**							<0.001	<0.001
**Insecta**								<0.001
**Vertebrata**								

Unpaired t-test showed that there was significant ($P<0.05$) difference in AT percent between different animal groups except between Mollusca and Echinodermata.
