# Supplementary material for: Unusually Long Palindromes Are Abundant in Mitochondrial Control Regions of Insects and Nematodes
Source: PLoS One. 2006 Dec 20;1(1):e110. doi: 10.1371/journal.pone.0000110 (PMC1762429; doi:10.1371/journal.pone.0000110)

CLUSTAL X - MULTIPLE SEQUENCE ALIGNMENT of short palindromes found in vertebrates

Bird species showing contraction of palindromes

Fish species showing contraction of palindrome

```
Gallus_sonneratii              ------TTTTTTAAAAACATTTTTTAAAAAA------  25
Gallus_lafayettei              ------TTTTTTAAAAACATTTTTTAAAAAA------  25
Gallus_gallus1                 ------TTTTTTAAAAACATTTTTTAAAAAA------  25
Syrmaticus_ellioti             ------TTTTTTAAAAACATTTTTTAAAAAA------  25
Gallus_varius1                 ATTTCGTTTTTTAAAAACATTTTTTAAAAAACTAAAT  37
Raja_porosa2                   -------------TAAGAATTCTTA------------  12
Mitu_tomentosa                 -----------CAAAAAAATTTTTTTG----------  16
Motacilla_madaraspatensis      --------------AATTAATATTAATT---------  14
Pseudobagrus_tenuis            ---------------ATTATGCATAAT----------  12
Gallus_varius2                 ----------------TTGTTAATTAACAA-------  14
Loxodonta_cyclotis             -----------------TGCTTATAAGCA--------  12
Sebastes_schlegeli             -----------AATACATATATGTATT----------  16
Cyprinodon_bovinus1            ---------ATAATACATATATGTATTAT--------  20
Diomedea_melanophris           -------------TACATTAATGTA------------  12
Thalassarche_steadi            -------------TACATTAATGTA------------  12
Jordanella_floridae            -------------TACATATATGTA------------  12
Cyprinodon_meeki3              -------------TACATATATGTA------------  12
Cyprinodon_atrorus2            -------------TACATATATGTA------------  12
Apodemus_mystacinus            -------------TACATATATGTA------------  12
Altolamprologus_calvus         -------------TACATATATGTA------------  12
Altolamprologus_compressiceps  -------------TACATATATGTA------------  12
ruler                          1.......10........20........30.......
```

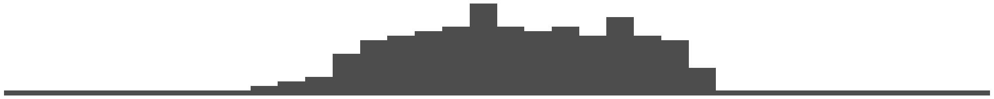

CLUSTAL X (1.81) MULTIPLE SEQUENCE ALIGNMENT of short and long palindomes found in veterbrates


```
             Thunnus_obesus -------------TACATATATGTA------------     12
              Milvus_milvus1 --------------ACATATATATGT-----------     12
            Zapus_hudsonius --------------ACATATATATGT-----------     12
       Phoxinus_oxycephalus2 -------------TACATATATATGTA----------     14
  Teratoscincus_keyserlingii2 ----------ATGTATATATATATATACAT-------     20
          Cyprinodon_meeki2 -------------GATAAATATTTATC----------     14
       Phoxinus_oxycephalus1 ------------TAACCTATAGGTTA-----------     14
             Gavia_stellata --------------TCACATATGTGA-----------     12
             Gavia_stellata --------------TCACATATGTGA-----------     12
       Phoxinus_oxycephalus3 ----------ATATGCGCATAT---------------     12
  Teratoscincus_keyserlingii1 ---------TATATACACATATGTGTATATA------     22
            Aquila_pomarina ------------ATATGTATACATAT-----------     14
              Aquila_clanga ------------ATATGTATACATAT-----------     14
          Aquila_nipalensis ------------ATATGTATACATAT-----------     14
        Porichthys_myriaster -------------TATGTACTAGTACATA--------     16
          Apeltes_quadracus ---------CATACATGTACTTCAAAGTACATGTATG     28
               Raja_porosa1 ----------TATATAGTACTATATA-----------     16
               Macaca_nigra ----------TATATAGTACTATATA-----------     16
        Macaca_fascicularis ---------ATATATAGTACTATATAT----------     18
           Macaca_arctoides ---------ATATATAGTACTATATAT----------     18
        Macaca_fascicularis ---------ATATATAGTACTATATAT----------     18
                      ruler 1.......10.........20.........30.......
```

Species of monkeys showing contraction of palindrome

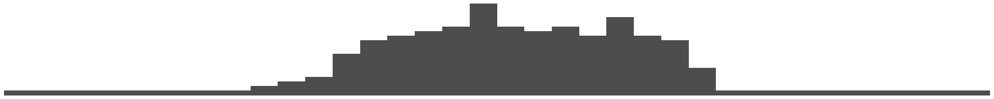

CLUSTAL X (1.81) MULTIPLE SEQUENCE ALIGNMENT of short and long palindomes found in veterbrates


```
                Ovis_aries ----------TATAAAGTACTTTATA-----------    16
     Latimeria_menadoensis --------------TAGTACGTACTA-----------    12
     Cyprinodon_fontinalis -------------TTAATATATATTAA----------    14
       Cyprinodon_atrorus1 -------------TTAATATATATTAA----------    14
        7Cyprinodon_meeki1 -------------TTAATATATATTAA----------    14
       Cyprinodon_bovinus2 -------------TTAATATATATTAA----------    14
         Megupsilon_aporus -------------TTAATATATATTAA----------    14
            Crax_globulosa ---------------CTCTCTAGAGAG----------    12
           Pauxi_unicornis ---------------CTCTCTAGAGAG----------    12
                 Mitu_mitu ---------------CTCTCTAGAGAG----------    12
         Nothocrax_urumutum ---------------CTCTCTAGAGAG----------    12
      Chamaepetes_goudotii ---------------CTCTCTAGAGAG----------    12
      Pungitius_pungitius ---------------TTCACGCGTGAA----------    12
                     ruler 1.......10........20........30.......
```

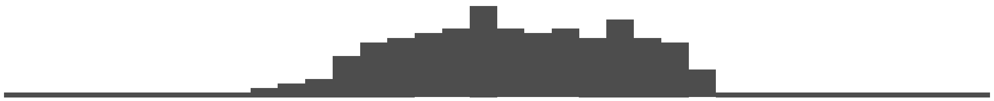

Supplement: Supplementary File S3 — CLUSTAL X - Multiple sequence alignment of short palindromes found in vertebrates (1.61 MB PDF) [file pone.0000110.s003.pdf]
